# Linear psoriasis unresponsive to multiple biological therapies (anti-TNF, anti-IL-12/23, and anti-IL-23): a case report^☆^

**DOI:** 10.1016/j.abd.2025.501237

**Published:** 2025-11-07

**Authors:** Sonia Romero-Romero, Maribel Iglesias-Sancho, Albert Martin-Poch, Montserrat Salleras-Redonnet

**Affiliations:** Department of Dermatology, Sagrat Cor University Hospital, Barcelona, Spain

*Dear Editor,*

Linear psoriasis (LP) is an exceptionally rare subtype of psoriasis that follows Blaschko’s lines. Based on its coexistence with non-segmental plaques of psoriasis vulgaris (PV), LP is classified as either the superimposed type (when it coexists with PV) or the isolated type (when it is the sole manifestation).^1^

We present the case of a 20-year-old Caucasian woman diagnosed with extensive psoriasis vulgaris at the age of 11, affecting all body areas. Initial treatments included topical methylprednisolone and narrowband UVB phototherapy with minimal improvement. At the age of 16, she started ustekinumab (Fig. 1), achieving near-complete remission, except for a persistent linear plaque on the posterior leg. The linear lesion showed a marked response to daily application of a fixed-dose combination of calcipotriol (50 μg/g) and betamethasone dipropionate (0.5 mg/g) for four weeks, resulting in near-complete resolution. However, when the regimen was changed to a maintenance schedule of twice-weekly application, the lesion recurred, although with reduced extent and severity.

**Fig. 1:**
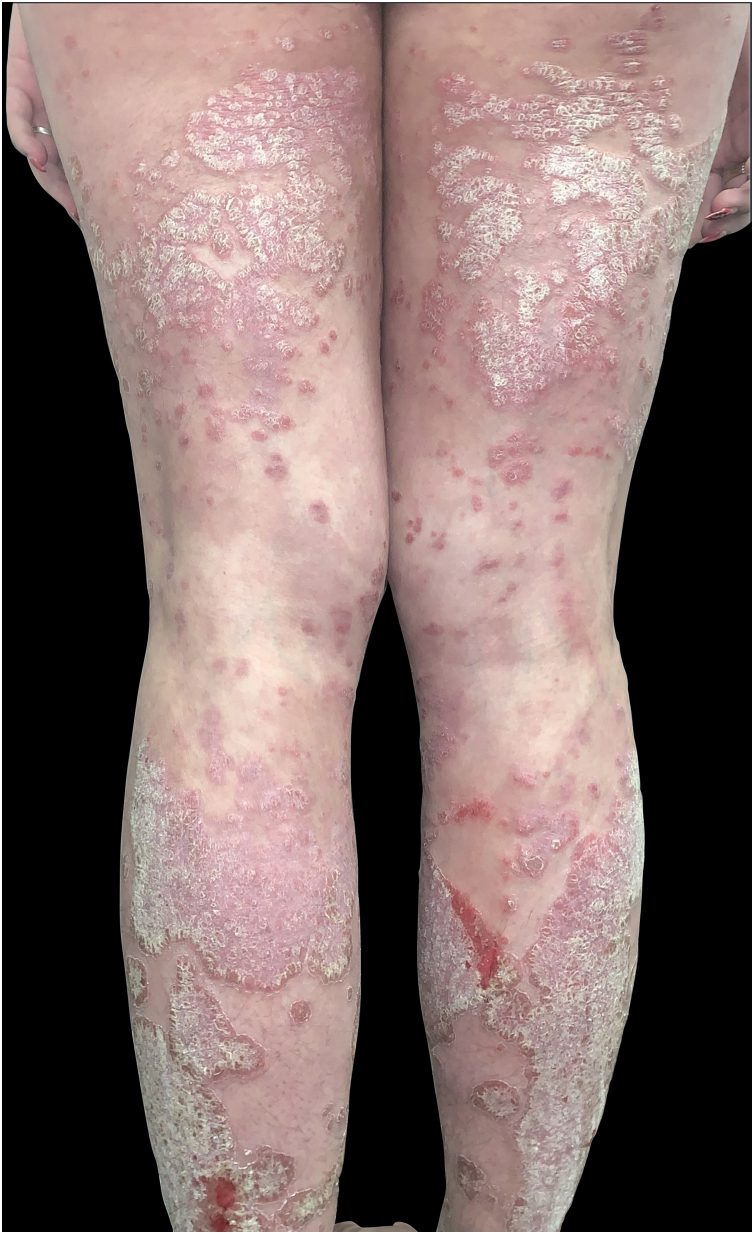
Patient with extensive plaque psoriasis prior to the initiation of biologic therapy with ustekinumab.

The treatment was discontinued for one year as the patient moved to another country and became pregnant. Upon her return, treatment with certolizumab was initiated, resulting in general clearance with persistence of the linear plaque. Postpartum exacerbation led to a switch to risankizumab, which cleared all lesions except the linear posterior leg plaque (Fig. 2). The clinical progression and characteristics of her lesions led to a diagnosis of linear psoriasis, unmasked by biological treatments.

**Fig. 2:**
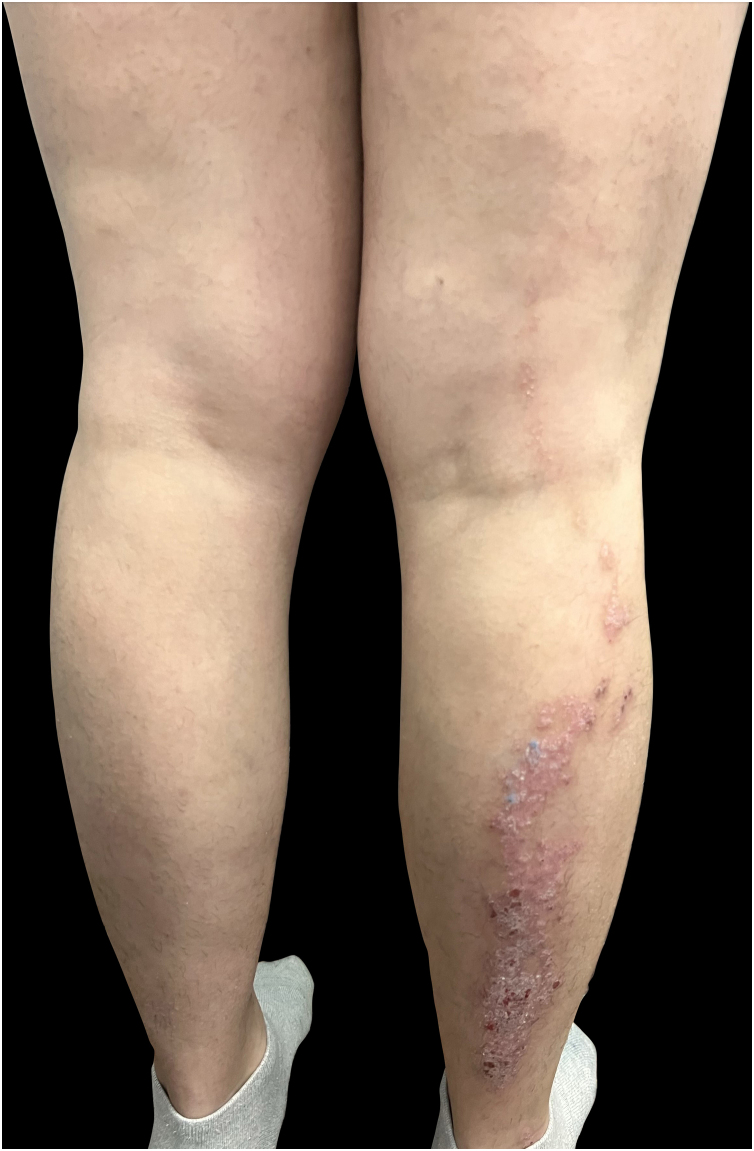
Patient receiving treatment with risankizumab, demonstrating persistence of a linear plaque following Blaschko’s lines.

Few cases of linear psoriasis have been reported in the literature, with superimposed LP type being more common than isolated LP type. The primary differential diagnosis for the isolated type is inflammatory linear verrucous epidermal nevus (ILVEN), which typically presents earlier with highly pruritic lesions that are refractory to treatment, unlike the generally asymptomatic lesions of LP.^2^, ^3^ Differentiating these conditions can be challenging due to their clinical and histological similarities. The presence of psoriasis in other regions can be pivotal in diagnosing LP.^3^ In cases of diagnostic uncertainty, a skin biopsy is recommended to distinguish linear psoriasis from ILVEN. Histopathologically, ILVEN is characterized by an alternating pattern of orthokeratosis and parakeratosis, often associated with granular layer changes, papillomatosis, and a chronic lymphomonocytic infiltrate. In contrast, linear psoriasis typically shows uniform psoriasiform hyperplasia, neutrophilic aggregates, and Munro microabscesses. Immunohistochemical markers can further aid in the distinction: ILVEN usually demonstrates low Ki-67 expression, normal keratin 10 levels, and absence of involucrin, whereas linear psoriasis is associated with high Ki-67 expression, reduced keratin 10, and presence of involucrin.^4^

With approximately 50 reported cases, the pathogenesis of LP remains poorly understood. However, genetic mosaicism ‒ specifically, postzygotic mutations occurring during embryogenesis leading to loss of heterozygosity (LOH) ‒ is suspected to play a key role. LOH may also account for the severity and chronicity of linear psoriatic lesions, as well as their resistance to antipsoriatic treatments.^3^, ^4^, ^5^ Postzygotic genetic alterations result in clones of keratinocytes predisposed to develop psoriatic lesions distributed along Blaschko’s lines. It is specifically proposed that somatic recombination is an underlying mechanism, potentially explaining the segmental distribution and treatment resistance often observed in these lesions.^6^

This case underscores the distinctive clinical challenge posed by LP, particularly when superimposed on PV. To date, only ten cases of LP treated with biological therapies have been documented, all of which involved LP superimposed on PV and were managed with anti-TNF agents or ustekinumab. Notably, LP exhibited refractoriness to anti-TNF therapy, whereas two of the three reported cases responded to Ustekinumab.^4^ To our knowledge, there is no available literature regarding the efficacy of alternative targeted therapies, such as anti-IL-23 or anti-IL-17 agents, in the management of LP.

We present the first reported case of superimposed LP demonstrating resistance to three distinct biological targets: anti-TNF, anti-IL-12/23, and anti-IL-23. The persistence of linear plaques despite advanced biological therapies suggests an intrinsic resistance, potentially associated with genetic mosaicism and loss of heterozygosity (LOH).

Resistance to ustekinumab ‒ the most effective therapy reported to date ‒ may suggest a potential cross-resistance to IL-23 inhibition. Nevertheless, based on our case and the existing literature, we believe that biological therapy may not be the most suitable treatment for this type of lesion. Further research is needed to elucidate the molecular mechanisms of LP and develop more effective treatments.

## ORCID IDs

Maribel Iglesias-Sancho: 0000-0001-8982-8522; Albert Martin-Poch: 0009-0003-9002-1779; Montserrat Salleras-Redonnet: 0000-0003-1004-2493

## Authors’ contributions

Sonia Romero-Romero: Manuscript writing and editing; Clinical review.

Maribel Iglesias-Sancho: Manuscript writing and editing; Clinical review.

Albert Martin-Poch: Critical review and revision of the manuscript.

Montserrat Salleras-Redonnet: Critical review and revision of the manuscript.

## Financial support

None declared.

## Research data availability

Does not apply.

## Conflicts of interest

None declared.
